# A Role for Dendritic Translation of CaMKIIα mRNA in Olfactory Plasticity

**DOI:** 10.1371/journal.pone.0040133

**Published:** 2012-06-29

**Authors:** Marie Néant-Fery, Eléonore Pérès, Carole Nasrallah, Monica Kessner, Simona Gribaudo, Charles Greer, Anne Didier, Alain Trembleau, Isabelle Caillé

**Affiliations:** 1 Team “Development and Plasticity of Neural Networks”, UPMC Univ Paris 06, UMR Centre National de la Recherche Scientifique 7102, Paris, France; 2 CNRS, UMR 7102, Paris, France; 3 Lyon Neuroscience Research Center, Neuroplasticity and Neuropathology of Olfactory Perception Team, Univ Lyon 1, Lyon, France; 4 Institut National Scientifique d’Études et de Recherches Médicales (INSERM), UMR 1028, Lyon, France; 5 CNRS, UMR5292, Lyon, France; 6 Department of Neurosurgery, Yale University School of Medicine, New Haven, Connecticut, United States of America; 7 University Paris Diderot, Sorbonne Paris Cité, Paris, France; Institut Curie, France

## Abstract

Local protein synthesis in dendrites contributes to the synaptic modifications underlying learning and memory. The mRNA encoding the α subunit of the calcium/calmodulin dependent Kinase II (CaMKIIα) is dendritically localized and locally translated. A role for CaMKIIα local translation in hippocampus-dependent memory has been demonstrated in mice with disrupted CaMKIIα dendritic translation, through deletion of CaMKIIα 3′UTR. We studied the dendritic localization and local translation of CaMKIIα in the mouse olfactory bulb (OB), the first relay of the olfactory pathway, which exhibits a high level of plasticity in response to olfactory experience. CaMKIIα is expressed by granule cells (GCs) of the OB. Through *in situ* hybridization and synaptosome preparation, we show that CaMKIIα mRNA is transported in GC dendrites, synaptically localized and might be locally translated at GC synapses. Increases in the synaptic localization of CaMKIIα mRNA and protein in response to brief exposure to new odors demonstrate that they are activity-dependent processes. The activity-induced dendritic transport of CaMKIIα mRNA can be inhibited by an NMDA receptor antagonist and mimicked by an NMDA receptor agonist. Finally, in mice devoid of CaMKIIα 3′UTR, the dendritic localization of CaMKIIα mRNA is disrupted in the OB and olfactory associative learning is severely impaired. Our studies thus reveal a new functional modality for CaMKIIα local translation, as an essential determinant of olfactory plasticity.

## Introduction

Since the seminal observation of polyribosomes localized at the base of dendritic spines [1], local translation in dendrites has been shown to be a major determinant of neuronal plasticity, participating in the synaptic changes that underlie learning and memory [2]. Among the mRNAs that have been clearly shown to be dendritically localized and locally translated is the mRNA encoding the α subunit of the calcium/calmodulin dependent Kinase II (CaMKIIα). CaMKII is a major component of postsynaptic densities (PSD) [3] and is essential to different forms of synaptic plasticity linked to learning and memory [4]. CaMKIIα mRNA is transported into dendrites of hippocampal and cortical neurons [5], [6] and this dendritic localization is mediated by its 3′UTR [7]. Local translation of CaMKIIα mRNA is found in biochemical fractions enriched for synapses (synaptosomes, SN) [8], [9] and in neuronal processes isolated from the soma of hippocampal neurons in culture [10]. In behaving animals, LTP induction in the hippocampus triggers a rapid delivery of CaMKIIα mRNA to dendrites [11] and synaptic sites [12]. Moreover, a 30 min exposure to light of dark-reared rats leads to an increase of CaMKIIα local translation in the visual cortex [13], [14], [15]. In *Drosophila*, neural activity drives CaMKIIα mRNA to synaptic sites, where it is rapidly translated and, most importantly, an olfactory associative learning task triggers an odor-specific induction of CaMKIIα mRNA synaptic transport and translation [16]. This strongly suggests that CamKIIα local translation is modulated by neural activity and contributes to the synaptic plasticity associated with learning and memory. To directly test the role of CamKIIα local translation in learning and memory, knocked-in mice were generated, in which CamKIIα 3′UTR was replaced by the 3′UTR of bovine growth hormone mRNA, a message that is not dendritically localized [17]. These mice display a dramatic reduction of CaMKIIα in PSDs of the hippocampus (HC), a reduction in late-phase long-term potentiation, and impairments of hippocampus-dependent memories. This confirms a role for CaMKIIα local translation in synaptic and behavioral plasticity.

We thus became interested in CaMKIIα local translation in the olfactory system. The olfactory bulb (OB) is the first relay of the olfactory pathway and presents a high level of plasticity in response to olfactory experience [18]. Here, we report that CaMKIIα is expressed by granule cells (GCs) of the OB. CaMKIIα mRNA is transported in GCs dendrites, synaptically localized and might be locally translated at GC synapses. This synaptic localization of CaMKIIα mRNA is regulated by olfactory activity through NMDAR. In mice devoid of CaMKIIα 3′UTR [17], the mRNA dendritic localization is dramatically decreased in the OB and olfactory associative learning is impaired. Our work thus suggests a fundamental role for CaMKIIα local translation in olfactory plasticity.

## Results

### CaMKIIα Expression in the OB

**Figure 1:**
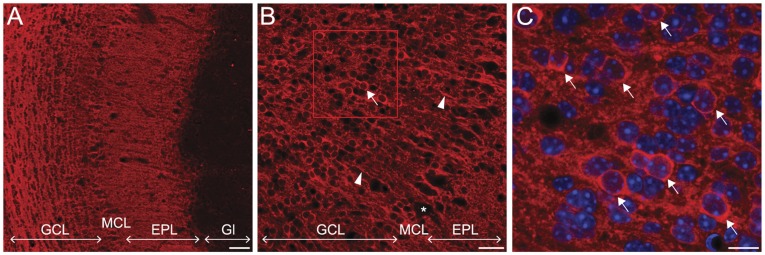
CaMKIIα expression in the OB. **A,B,C** Immunostaining for CaMKIIα (red) on olfactory bulb (OB) sections. **A:** CaMKIIα immunoreactivity is present in the Granule Cell Layer (GCL) and in the External Plexiform Layer (EPL), where granule cells (GCs) extend their apical dendrites. The glomerular layer (Gl) appears unlabeled. Scale bar: 50 µm. **B,** Higher magnification of the GCL shows staining of some GCs in their cell bodies (arrow) and in their dendrites extending towards the EPL (arrowheads). Mitral cells do not express the protein (star). Scale bar: 25 µm. **C,** Magnification of the boxed region in B. Some GCs express CaMKIIα in the thin rim of cytoplasm surrounding their nuclei (arrows). Nuclei are counterstained with DAPI. Scale bar: 10 µm.

We first investigated the expression pattern of CaMKIIα in the OB by immunohistochemistry. We observed a strong labeling in the granule cell layer (GCL) and the external plexiform layer (EPL) (Fig. 1A). The GCL contains the cell bodies of GCs, which extend their long apical dendrite into the EPL, where they synapse onto the dendrites of mitral cells, forming reciprocal dendro-dendritic synapses. The staining was absent in the glomerular layer (Fig 1A). At the cellular level, CaMKIIα immunoreactivity surrounds GCs nuclei in their thin rim of cytoplasm and can occasionally be observed in their dendrites, extending towards the EPL (Fig. 1B and C). In the EPL, however, dendrites could not be individualized and CaMKIIα staining appeared blurry. Mitral cells (MCs), which form a single cell layer composed of larger cells around the GCL, appeared unlabeled (Fig. 1B). Overall, this pattern of immunoreactivity is consistent with previous work [19] and confirms that CaMKIIα is expressed by GCs in the OB and present in both their cell bodies and dendrites.

### CaMKIIα mRNA Dendritic Localization and Local Translation in GCs

**Figure 2:**
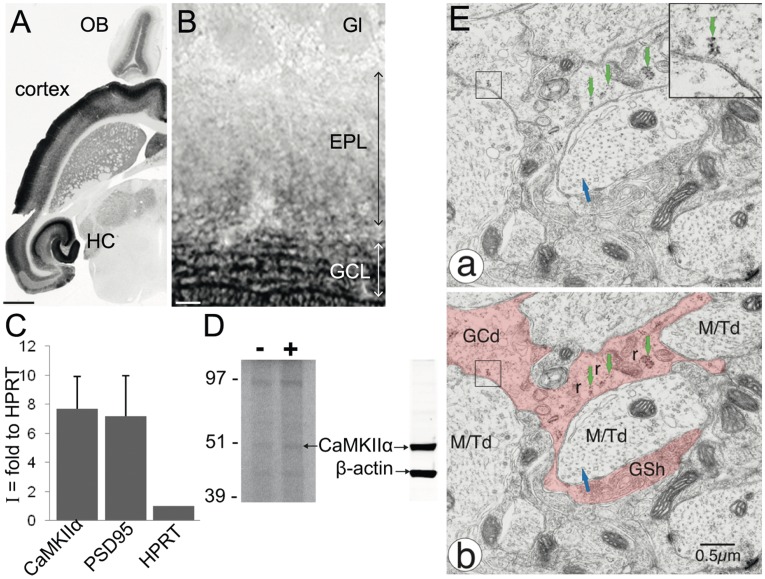
CaMKIIα mRNA dendritic localization and translatability. **A,B** *In situ* hybridization for CaMKIIα mRNA. **A,** On a horizontal section, CaMKIIα mRNA is visible in multiple regions of the brain, particularly in the hippocampus (HC), cortex and olfactory bulb (OB). Scale bar: 500 µm. **B,** Higher magnification of the OB shows strong CaMKIIα mRNA expression in the granule cell layer (GCL) and in a diffuse staining of the external plexiform layer (EPL), where GCs extend their apical dendrites. Gl: glomerular layer. Scale bar: 25 µm. **C,** Quantification of mRNA levels in synaptosomes (SN) by quantitative PCR. CaMKIIα and PSD95 transcripts are highly elevated (7 fold) at the synaptic level, as compared to HPRT mRNA, a transcript restricted to the soma. “I” is the index of synaptic localization and represents the ratio of mRNA present in the SN fraction over the total quantity present in the OB homogenate, normalized to HPRT mRNA. Error bars represent sem (n = 3). **D,** SN were metabolically labeled with a mixture of ^35^S-Met and ^35^S-Cys with (lane +) or without (lane -) stimulation by 10 µM glutamate and 50 µM NMDA. Newly-synthesized proteins were detected by autoradiography (left panel) or blotted with an anti-CaMKIIα antibody (right panel). **Ea,b** Electron micrograph of a dendrodendritic synapse between a granule cell dendrite (GCd) and a mitral/tufted cell dendrite (M/Td). Ribosomes (r, arrows) are located in the GCd, at the base of, or in close proximity to, a spine. The inset displays a magnification of the boxed region showing polyribosomes with a characteristic rosette morphology. The GC is colorized in pink in b, and the blue arrow in the granule cell spine head (GSh) indicates the synapse formed onto the mitral/tufted cell dendrite (M/Td). Scale bar: 0.5 µm.

As CaMKIIα mRNA has been described to be dendritically localized in the cortex and HC [5], [6], we investigated its localization in the OB. *In situ* hybridization (ISH) against CaMKIIα mRNA shows strong staining in multiple regions of the brain, as described (Fig. 2A). Staining in the HC is in agreement with previous reports: in the dentate gyrus and CA1-CA3, cell bodies are strongly labeled and a more diffuse staining is observed in the dendritic compartments. In the OB, the GCL is strongly stained. Higher magnification of the OB confirms a strong expression of CaMKIIα mRNA in the GCL and reveals a diffuse staining in the EPL, where the GC apical dendrites arborize (Fig. 2B). This suggests that CaMKIIα mRNA is dendritically localized in GCs.

To confirm and refine this result, we prepared OB synaptosomes (SN). SN are isolated resealed-synapses obtained by a biochemical fractionation. mRNAs extracted from these preparations were retro-transcribed and analyzed by quantitative PCR to calculate an index of synaptic localization “I”. For a given mRNA, this index is the ratio of the quantity of synaptic mRNA over the quantity of this mRNA in total brain extract normalized to HPRT, a transcript restricted to the cell soma. In all analyzed experiments, HPRT mRNA contamination in SN preparation was between 0.1 and 5% (not shown). With this technique, we found that CaMKIIα mRNA is highly enriched in SN (7-fold to HPRT, n = 3), suggesting synaptic localization (Fig. 2C). PSD95 mRNA is known to be synaptically localized [20], [21] and was included as a positive control. Its index of synaptic localization was similar that of CaMKIIα mRNA. Taken together, these results strongly suggest that CaMKIIα mRNA is dendritically and synaptically localized in GCs.

We then assessed whether the synaptically localized CaMKIIα mRNA could be locally translated. To this extent, we prepared SN and metabolically labeled them with a mixture of ^35^S-Met and ^35^S-Cys with or without stimulation by 10 µM glutamate and 50 µM NMDA (Fig.2D). SDS-PAGE and autoradiography of the same quantity of proteins from unactivated or activated SN showed a global 1.7 increase of protein synthesis with stimulation (n = 3, p<0.05). Among the metabolically labeled proteins, one had a size corresponding to CaMKIIα, as revealed by Western Blot and its quantity similarly increased by 1.6 upon Glu/NMDA stimulation. This suggests that CaMKIIα might be newly-synthesized in our SN preparation and thus that the synaptically localized CaMKIIα mRNA might be locally translated.

Synaptic local translation requires that functional translation machinery is located close to the synapse. The association of polyribosomes with dendritic spines of granule cells of the dendate gyrus has been clearly documented by electron microscopy [1]. We thus verified by EM that some polyribosomes could also be found associated with GCs spines forming dendrodendritic synapses in the OB. Indeed, we found polyribosomal rosettes at the base of, or in close proximity to, the spine neck (Fig. 2E), similar to those observed in the HC. In addition, we quantified EM sections of GCs dendrites in the EPL for the density of ribosome clusters (ranging in size from 2 to 20) along with the density of synaptic appositions. We found that the density of ribosome clusters correlated with the density of synapses (mean number of ribosomes 6.79+/−0.66 per 80 µm^2^ versus mean number of synapses 8.76+/−0.45 per 80 µm^2^, Spearman’s correlation r = 0.55, p<0.0008, n = 33 fields of 80 µm^2^). This correlation suggests that the presence of a translational machinery in GCs dendrites depends on the presence of synapses, raising the possibility that GCs dendrites are functional for synaptic local translation.

### CaMKIIα mRNA Synaptic Localization is Regulated by Olfactory Activity through NMDAR

In hippocampal and cortical neurons, CaMKIIα mRNA is dendritically localized and locally translated in response to synaptic stimulation [8], [9], [10], [11], [12], [13], [15], [16], [22].

**Figure 3:**
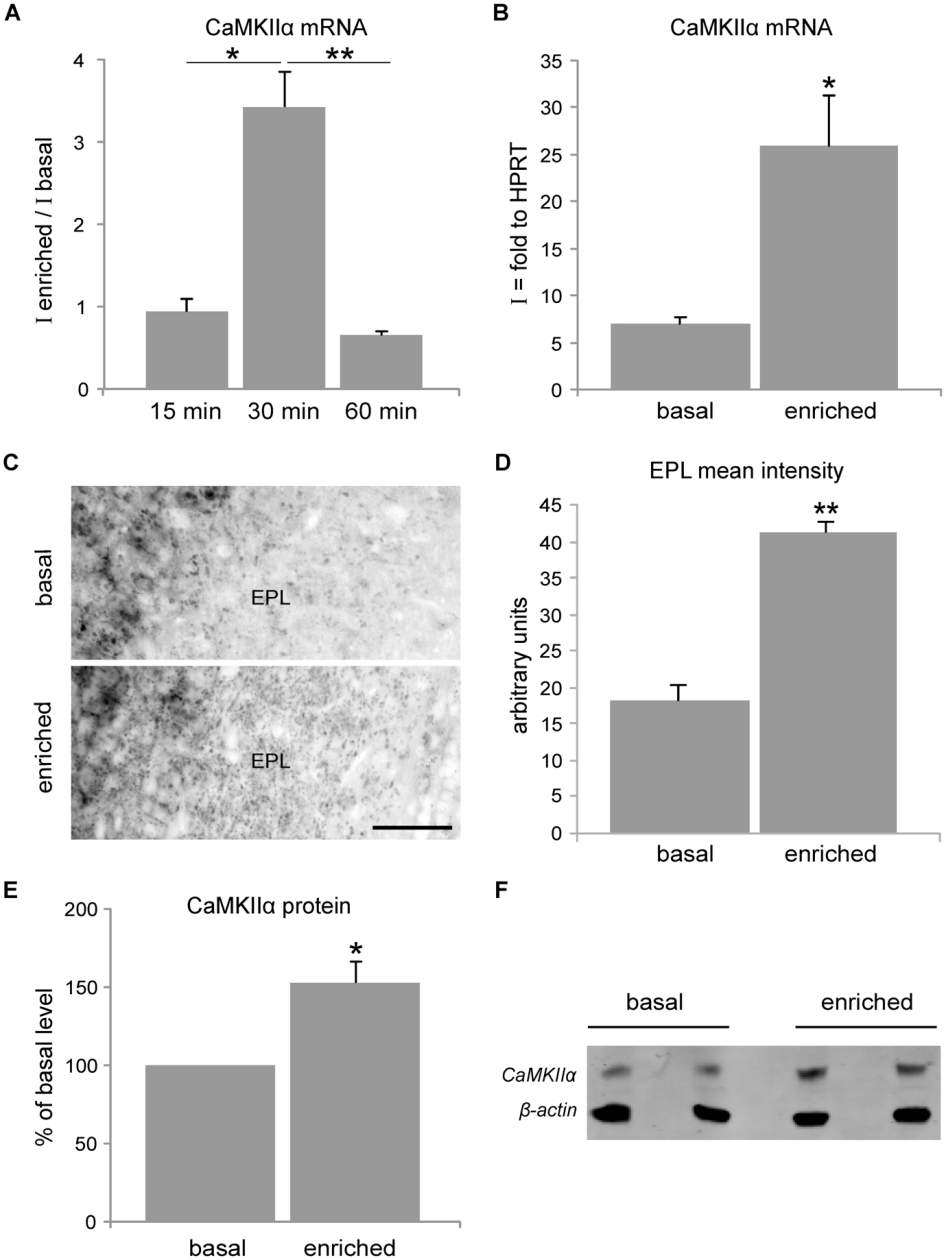
CaMKIIα mRNA synaptic localization is regulated by olfactory activity. **A,** Mice were presented with a tea-ball containing either garlic and tarragon (“enriched”) or nothing (“basal”) for various durations. 30 min of this olfactory enrichment raises the levels of CaMKIIα mRNA in synaptosomes (SN) by 3.4-fold as compared to basal levels. No increase is observed after 15 min of exposure, and mRNA levels return to baseline (index ratio of 1) 60 min after exposure. “I” is the index of synaptic localization and represents the ratio of mRNA present in the SN fraction over the total quantity present in the OB homogenate. Error bars represent sem. (n = 3 for 15 min and 30 min, n = 2 for 60 min; *p = 0.005, **p<0.02, t-test) **B,** CaMKIIα index of synaptic localization rises from 7 in basal conditions to 26 upon 30 min of olfactory enrichment (n = 3; *p<0.02, t-test). **C, D** *In situ* hybridization for CaMKIIα mRNA of control or 30 min enriched mice shows an increase of the EPL staining upon enrichment Scale bar: 50 µm. Quantification of the signal showed that it was increased by 2.3 upon enrichment (n = 2, p = 0.006). **E,F** Western blot of SN shows a 1.5 increase in CaMKIIα protein in enriched conditions versus basal conditions (n = 4, p<0.05). The results were normalized over β-actin signal.

We thus tested the effects of olfactory activity on CaMKIIα dendritic localization in the OB through a simple olfactory enrichment protocol. Groups of 10 mice were first habituated to a clean new environment for one hr. They were then presented for 15, 30 or 60 min with a tea-ball containing a cocktail of unfamiliar odors (garlic and tarragon). Control groups were presented with an empty tea-ball and represent the basal conditions. SN were then prepared and mRNAs quantified, as described above. After 15 min of enrichment, we found no increase in the quantity of CaMKIIα mRNA in SN as compared to basal conditions (Fig. 3A). However, after 30 min of enrichment, CaMKIIα mRNA levels in SN were dramatically increased by 3.4, as compared to basal conditions (Fig. 3A), with an index of synaptic localization rising from 7 in basal conditions to 26 in enriched conditions (n = 3; p<0.02, t-test) (Fig. 3B). After one hr of enrichment, CaMKIIα mRNA levels in SN returned to basal levels (Fig. 3A). Enrichment had no effect on CaMKIIα transcription, as CaMKIIα mRNA levels in total OB extracts remained unchanged (data not shown). These results suggest an activity-dependent re-localization of CaMKIIα mRNA in GCs dendrites, during a restricted time-window of olfactory enrichment.

We then verified whether this increase in dendritic CaMKIIα mRNA could be visualized by ISH. Indeed, the dendritic staining in the EPL appeared more intense after a 30 min enrichment (Fig. 3C). Quantification of the signal showed that it was increased by 2.3 upon enrichment (Fig.3D, n = 2, p = 0.006).

To test whether the increased dendritic CaMKIIα mRNA could lead to its increased local translation, we quantified CaMKIIα protein in SN after enrichment and saw that there was an increase of 1.5 as compared to basal conditions (n = 4, p<0.05) (Fig. 3E). This could be the consequence of an increased local translation of CaMKIIα mRNA in some GC spines.

**Figure 4:**
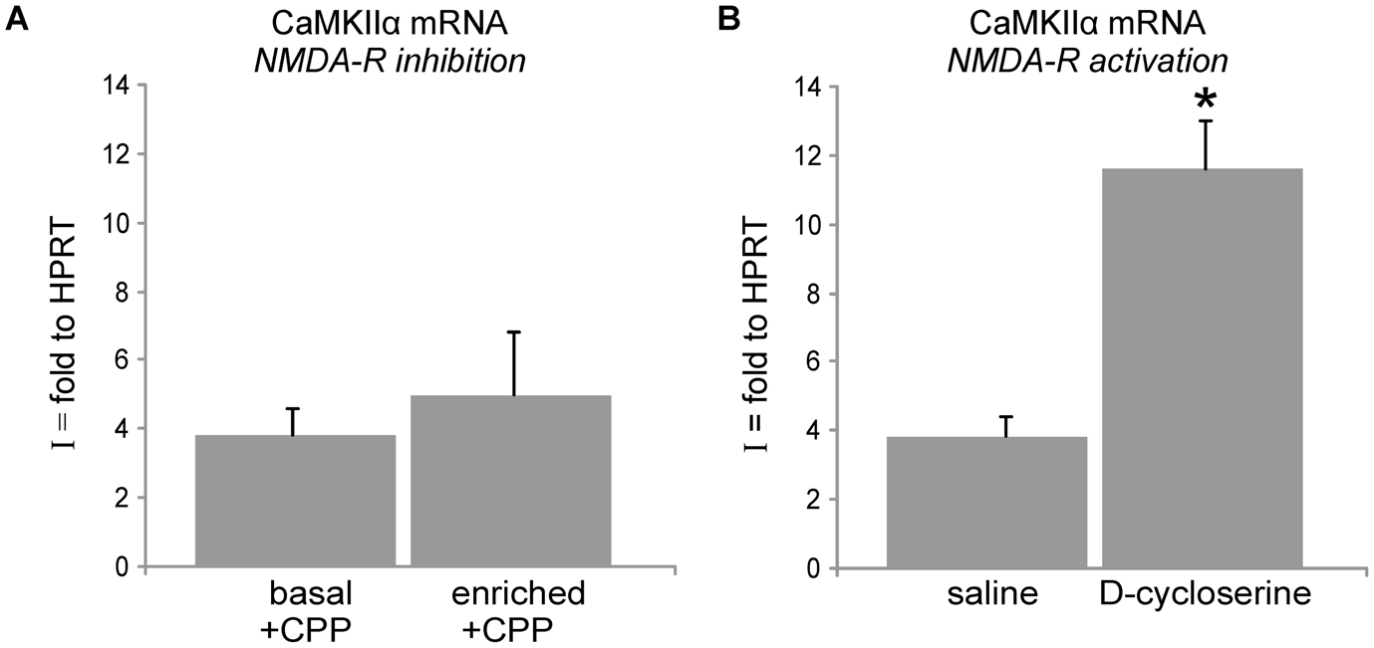
CaMKIIα mRNA synaptic localization is regulated by NMDAR. **A,** NMDAR antagonist: 30 min prior to the 30 min olfactory enrichment protocol, mice were injected with CPP. Upon 30 min of olfactory enrichment, the synaptic localization index remains unchanged as compared to basal conditions. NMDA receptor blockade thus prevents the increase in CaMKIIα mRNA observed upon olfactory enrichment (compare with B) (n = 3; p = 0.6, t-test). **B,** NMDAR agonist: mice were injected with D-cycloserine or saline. 30 min after injection, the index of synaptic localization is increased 3.4-fold as compared to saline, thus recapitulating the effect of olfactory enrichment on CaMKIIα mRNA localization (compare with B) (n = 2 for saline, n = 3 for D-cycloserine; *p = 0.02, t-test).

NMDA receptors are pivotal in activity-dependent mRNA synaptic localization [23]. Moreover, they are essential to the dendrodendritic inhibition exerted by GCs onto mitral cells [24], [25]. To test their role in the activity-dependent dendritic localization of CaMKIIα mRNA in the OB, we injected mice with CPP ((RS)-3-(2-Carboxypiperazin-4-yl)-propyl-1-phosphonic acid, 10 mg/kg by intraperitoneal injection), an NMDA-receptor antagonist, 30 min prior to olfactory enrichment. In these conditions, 30 min of olfactory enrichment had no effect on synaptic CaMKIIα mRNA levels that remained at basal level in SN (n = 3; p = 0.6, t-test) (Fig. 4A), contrary to what is seen in untreated animals (Fig.3B). NMDA-receptors blockade thus prevents the activity-induced increase of CaMKIIα mRNA levels in SN. To fully test the role of NMDA-receptors in the dendritic localization of CaMKIIα mRNA in the OB, we treated mice with D-cycloserine ((R)-4-Amino-3-isoxazolidone, 4-Amino-3-isoxazolidinone, 20 mg/kg by intraperitoneal injection), an agonist of NMDA receptors, 30 min before sacrifice. Control mice were injected with saline. In D-cycloserine injected mice, CaMKIIα mRNA levels in SN were increased 3.4 fold as compared to control (n = 3; p = 0.02, t-test) (Fig.4B). Activating NMDA-receptors through the use of D-cycloserine thus recapitulates the increase in CaMKIIα mRNA levels in SN, consecutive to a 30 min olfactory enrichment. Neither CPP nor D-cycloserine injections affected levels of CaMKIIα mRNA in total OB extracts (data not shown). Our data strongly suggests that olfactory activity regulates CaMKIIα mRNA synaptic localization in GCs through NMDA receptors.

### Altered CaMKIIα mRNA Dendritic Localization Disrupts Olfactory Associative Learning

**Figure 5:**
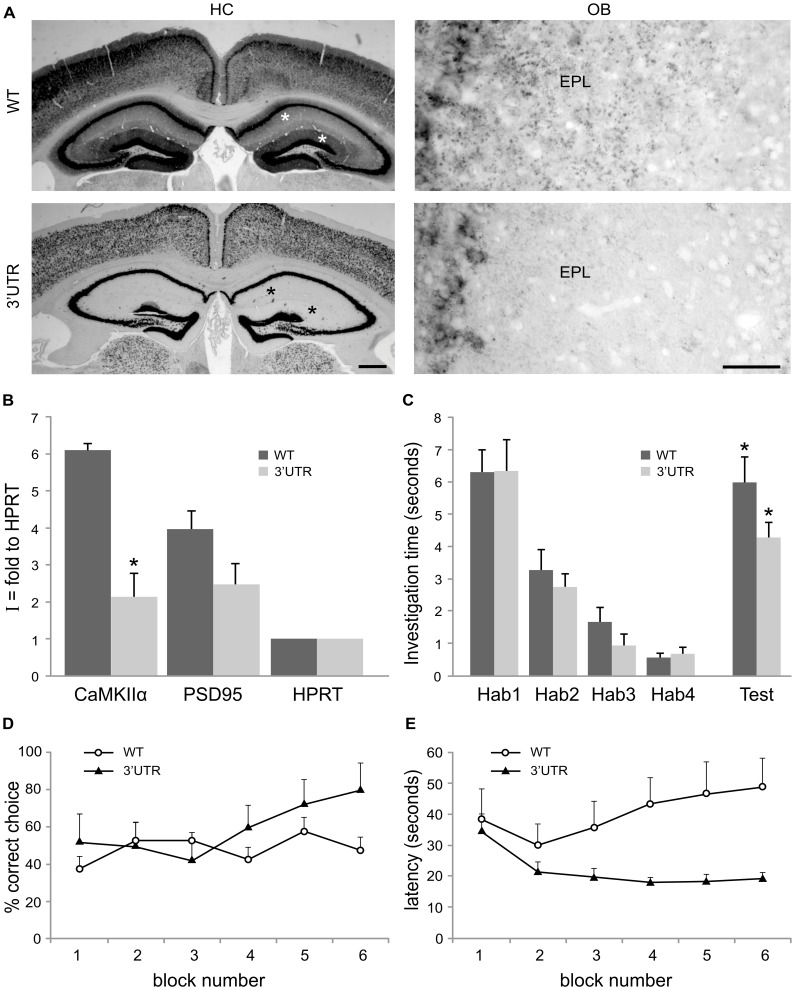
Altered CaMKIIα mRNA and protein synaptic localization disrupts olfactory associative learning. **A,** *In situ* hybridization for CaMKIIα mRNA in wild-type mice (WT, upper panels) and 3′UTR mutants (3′UTR, lower panels) shows a dramatic decrease of the dendritic staining (stars) in the mutant hippocampus (HC, left panels) and olfactory bulb (OB, right panels). EPL: external plexiform layer Scale bars: 200 µm and 50 µm, in HC and OB respectively. **B,** Quantification of the index of synaptic localization (I) of CaMKIIα and PSD95 mRNAs normalized to HPRT mRNA in WT and 3′UTR mutants. CaMKIIα mRNA index is significantly decreased in 3′UTR mutants as compared to WT (n = 3, p = 0.026) **C,** Behavioral habituation/dishabituation paradigm: both WT and 3′UTR mutants mice habituated to the habituation odor, as shown by decreased investigation time across the four trials (Hab1-Hab4) (WT: F(3,27) = 30.452, p<0.001; 3′UTR: F(3,27) = 20.469, p<0.001; ANOVA, trial effect) and discriminated. Upon presentation of the test odor (Test), the investigation time increased when compared to Hab4, indicating that the animals could discriminate the test odor from the habituation odor (WT: *p<0.00005; 3′UTR: *p<0.00005, t-test). **D,** Olfactory associative learning: mice were conditioned to associate an odor stimulus to a food reward. Success rate results are presented as the percentage of correct choice across consecutive blocks of trials (1 block = 4 trials). Conditioned WT mice learned the task as indicated by the increase in correct choice throughout blocks (F(5,49) = 3.391, p<0.01; ANOVA, block effect). In contrast, conditioned 3′UTR mutants did not show an increase in success rate across trials (F(5,54) = 1.078, p>0.1; ANOVA, block effect), showing that impaired CaMKIIα mRNA dendritic localization disrupts olfactory-associative learning. **E,** Latencies: conditioned WT mice showed a decrease in latency confirming that they learned the task (F(5,49) = 4.037, p<0.005; ANOVA, block effect). In contrast, latencies of conditioned 3′UTR mutants did not significantly change (F(5,54) = 0.635, p>0.5; ANOVA, block effect), indicating that the mutants were not able to associate odor and reward.

To test a role of CaMKIIα local translation in olfactory functions, we took advantage of the knocked-in mice where CaMKIIα 3′UTR has been replaced by the 3′UTR of an unlocalized mRNA [17]. By ISH, we found strongly reduced dendritic labeling in the HC of the 3′UTR mutants as described, and also observed a similar reduction in the OB (Fig. 5A). Quantification of this staining in the EPL showed that it was reduced by 4 in the mutants (WT  = 18,1±1.6; 3′UTR  = 4.4±1.3, mean intensity ± sem, n = 2, p = 0,02).

This decreased localization of CaMKIIα mRNA in OB dendrites was confirmed through SN preparation followed by RTqPCR: the index of synaptic localization for CaMKIIα mRNA normalized over HPRT was reduced by 65% in the 3′UTR mutants as compared to the WT (3′UTR, I = 2.1±0.6 versus WT, I = 6±0.18, p = 0.026, n = 3) (Fig.5B), whereas this index was not significantly changed for PSD95 mRNA.

We then tested the olfactory capacity of the mutants. First we used the habituation/dishabituation paradigm to assess olfactory habituation and discrimination between two odorants (Carvone and Pentanol). When presented with the habituation odorant, both control and mutant mice investigation time decreased across the four habituation trials (control: F(3,27) = 30.452, p<0.001; mutant: F(3,27) = 20.469, p<0.001; trial effect) (Fig. 5C). When the test odor was then presented to the mice, the time of odor investigation significantly increased compared to the last habituation trial (Hab4) in both groups of mice (control: p<0.00005; mutant: p<0.00005, t-test) (Fig.5C). These results indicate that the mice detected the odor, habituated to it and were able to discriminate the test odor from the habituation one, regardless of their genotype. Taken together, these data indicate that the mutant mice have normal basic olfactory function.

In order to test if the reduction of CaMKIIα mRNA in dendrites affects olfactory learning capacity, mice were submitted to an olfactory associative learning task. The mice were trained to use an olfactory cue to find a reward hidden in a hole (see Material and Methods). This paradigm was chosen because it is hippocampus-independent [26]. The percentage of correct choice in the control group increased significantly across blocks (one block  = 4 trials) (F(5,49) = 3.391, p<0.01; ANOVA, block effect) (Fig. 5D). This indicates that control mice learn to associate the odorant to the reward. Learning was confirmed by the decrease in latency across trials (F(5,49) = 4.037, p<0.005; ANOVA, block effect) (Fig. 5E). In contrast, the success rate of the mutants did not increase with blocks, nor did the latency decrease (success rate: F(5,54) = 1.078, p>0.1; latency: F(5,54) = 0.635, p>0.5; ANOVA, block effect) (Fig. 4D and E). A direct group comparison confirmed that the two groups of mice behaved differently (success rate: F(1,103) = 7.43, p<0.01; latency: F(1,103) = 20.571, p<0.001; ANOVA, group effect).

This indicates that the 3′UTR mutants could not learn the odor-reward association, and suggests a role for CaMKIIα mRNA dendritic localization and local translation in olfactory associative learning.

## Discussion

### CaMKIIα Localization in the OB

Our immunocytochemical localization of CaMKIIα shows that CaMKIIα is exclusively expressed by GCs in the OB. As described before [19], CaMKIIα is not expressed by glutamatergic mitral cells, whereas it is typically associated with glutamatergic synapses in other brain regions. The immunostaining in the EPL suggests its presence in the dendro-dendritic reciprocal synapses between GC dendritic spines and mitral cells, which was indeed previously observed by immuno-electron microscopy [19].

Interestingly, we could also detect CaMKIIα mRNA in the EPL. CaMKIIα mRNA has been among the first mRNAs to be described in dendrites of the HC and cortex [5], [6], but the authors did not address localization in the OB. By ISH, we could clearly see CaMKIIα strong expression in cell bodies of the GCL and a lighter staining extending in a gradient into the EPL, where GCs project their apical dendrites. This dendritic labeling appears to be specific, as it disappears in the knocked-in mice, where CaMKIIα 3′UTR is ablated [17] (see Fig. 5A).

To confirm and quantify CaMKIIα mRNA synaptic localization, we performed RTqPCR on mRNAs from OB SN. The index of synaptic localization was indeed 7 times higher than HPRT mRNA, an unlocalized mRNA. This index was similar to the one we quantified in the HC (not shown) and to previous studies [12], [27], thus validating our SN preparation for the analysis of CaMKIIα mRNA localization. Of importance, the synaptically localized CaMKIIα mRNA in the OB might be translated, since we could metabolically label newly-synthesized CaMKIIα protein in SN (see Fig. 2D). Accordingly, our EM observation showed that polyribosome could be found in GCs dendritic shaft in close proximity to spines forming dendro-dendritic synapse and that the number of polyribosomes in GCs dendrites is correlated to the number of synaptic appositions. This is consistent with previous observations made in other systems [1] and suggests for the first time that GCs synapses are functional for local translation.

### Activity-dependent Transport and Translation of CaMKIIα mRNA in the OB

Here, we show that synaptic CaMKIIα mRNA is dramatically increased by a 30 min exposure to new odors. In cultures of hippocampal neurons, depolarization increases the motility of CaMKIIα mRNA containing granules [28], [29], [30]. In the HC of freely moving rats, LTP induction triggers delivery of CaMKIIα mRNA in dendrites [11] and synaptic sites [12]. Contrary to what we observe in GC dendrites, this transport appeared to be NMDA independent [12]. This might reflect a difference in the regulation of CaMKIIα mRNA transport between OB and HC but also a difference in the timing of CPP administration (30 min before enrichment in our study versus 2 hrs in the HC study). In our experiment, NMDA proves to be necessary and sufficient to support the increase of synaptic CaMKIIα mRNA localization induced by olfactory enrichment. Interestingly, glutamate acting through NMDA-receptors is essential to the proper functioning of dendrodendritic synapses, as the release of GABA from GCs spines depends on NMDAR activation [24], [25]. CaMKIIα mRNA transport and local translation might play a role in this process, as CaMKII can regulate neurotransmitter release at presynaptic sites [5] and as NMDAR can be phosphorylated by CaMKII [31].

The increased transport of CaMKIIα mRNA upon olfactory enrichment is accompanied by a 1.5 fold-increase in synaptic CaMKIIα protein. This suggests an increase in CaMKIIα mRNA local translation upon olfactory enrichment. This increase is reminiscent of what was observed in the visual cortex of dark-reared rats exposed to light for 30 min [13]. Interestingly, the response in visual cortex was also NMDAR dependent, since it was inhibited by CPP [15]. An NMDA-dependence of CaMKIIα mRNA local translation has also been observed *ex vivo* in SN [8], [9].

### CaMKIIα mRNA Local Translation is Necessary for Olfactory Associative Learning

We analyzed the function of CaMKIIα mRNA local translation in the olfactory pathway using mutant mice expressing a form of CaMKIIα mRNA without its 3′UTR [17]. These mice were originally created to analyze the functional role of CaMKIIα mRNA local translation in the HC and showed its importance in synaptic and behavioral plasticity. The mice displayed a reduction in late-phase LTP and impairments in spatial memory, associative fear conditioning and object recognition memory. The authors reported a massive reduction of CaMKIIα mRNA in dendrites and SN from the HC, which we also observe in the OB. In this initial study, an unexpected 50% reduction of CaMKIIα protein in whole hippocampal extracts was also observed in the mutants. We observe a similar reduction in the OB (not shown). One can thus not formally exclude that the phenotype we observe could be partly due to this reduction.

In a subsequent article concerning the same mice [32], quantitative proteomics of HC synapses showed that the synaptic protein constituents were not substantially altered in the mutant mice, apart from CaMKIIα itself. Of importance, in the OB, we see that the GCL size is unaltered in the mutants, suggesting that GCs survival is normal. Moreover, in an olfactory habituation/dishabituation test (Fig. 5C) [33], the mutant mice displayed normal basic olfactory function in terms of detection, habituation and discriminations of odors. Together, these data suggest that the reduction of CaMKIIα mRNA local translation does not trigger major compensatory changes that could affect the OB functioning. Nevertheless, we report here that, in an olfactory associative learning paradigm [33], [34], [35], these mice were incapable of learning. Noticeably, the HC is not required for acquisition of this type of olfactory non-spatial associative task [26], [36].

The importance of CaMKII mRNA local translation during associative olfactory learning was previously noted in *Drosophila* [16]. Indeed, using fluorescent reporters of translation, the induction of synaptic CaMKII synthesis was observed in several brain centers following a training paradigm of repetitive odor, paired with electric shock. Remarkably, this synaptic induction was odor specific. This drew a strong correlation between CaMKII local translation and the synaptic changes underlying olfactory learning and memory in *Drosophila*.

Our results confirm and extend these data to the mammalian olfactory pathway. The 3′UTR mutant mice could not learn to associate an odor with a reward, which indicates that CaMKIIα local translation is essential to the synaptic modifications associated with olfactory associative learning. This defective learning might be the consequence of disrupted CaMKIIα local translation in multiple regions of the olfactory pathway. Among them, the OB is likely to play an essential role since i) we show an activity-regulated CaMKIIα mRNA transport and translation in the OB and ii) olfactory learning-induced changes can occur within the OB itself. For instance, the oscillatory response of the whole OB network evolves with learning, in close correlation with behavioral performances [37]. In addition, the response of MCs to an odor depends on the odor association with a positive or negative reinforcement [38]. Most importantly, the synchronized firing of MCs during olfactory associative learning conveys information on the odor association to a reward, so that information on stimulus reward is encoded within the OB itself [39]. The neural representation of the odors in the OB is thus highly modulated by olfactory learning [18] and CaMKIIα local translation might play a particularly meaningful role in the synaptic modifications underlying these changes.

Finally, GCs undergo an extreme form of plasticity, since they are constantly renewed throughout life. We have recently shown that the Fragile X Mental Retardation Protein (FMRP) is a master regulator of neo-GCs morphogenesis [40]. Interestingly, CaMKIIα mRNA is one of FMRP’s mRNA targets [41]. In addition to regulating synaptic strength, CaMKIIα regulates structural plasticity by controlling spine size and density [42], [43], [44], activity-dependent filopodia growth and spine formation [45] and by stabilizing dendritic arbor structure [14]. It is thus tempting to speculate that CaMKIIα local translation might also play a fundamental role in new GCs integration.

## Materials and Methods

### Animals

C57Bl6, CD1 or 3′UTR mutants and control littermates (in a C57bl6 genetic background) 2-month old males were used in all experiments. This study was carried out in strict accordance with the recommendations of the CNRS «Formation à l’Expérimentation Animale ». The protocol was approved by the Comité Régional d’Ethique en Expérimentation Animale N°3 of the région Ile de France (File number p3/2008/047).

### In Situ Hybridization

20 µm cryostat sections of olfactory bulb (OB) were hybridized with a Digoxygenin-labeled CaMKIIα antisense probe (GenBank accession number NM_177407, nucleotides 111–1619) as previously described [20].

For quantification, photomicrographs were taken at a magnification of 40X and the intensity of labeling was quantified with ImageJ on defined surfaces of the EPL and normalized to unstained regions of the periglomerular area.

### CaMKIIα Immunohistochemistry

50 µm OB vibratome sections were saturated for 1 h at room-temperature in a blocking buffer (BB: PBS, 10% fœtal bovine serum, 0.5-1% triton-X). Sections were incubated at 4°C with an anti-CaMKIIα antibody (mouse monoclonal, 1∶200; Pierce Antibodies) for 48 h in BB, rinsed in PBS, and revealed by an anti-mouse DyLight 549 secondary antibody (1∶2000; Jackson Immunoresearch) in BB for 1 h at room-temperature. Acquisitions were made on an SP5 Leica confocal microscope.

### Synaptosome Preparation for RNA Analysis

Synaptosomes were isolated as described previously [27] with some modifications. Briefly, the OBs from 10 adult mice per condition were homogenized in 9 ml of 320 mM sucrose, 1 mM EDTA, supplemented with protease (Complete tablets, Roche) and RNase inhibitors (20 U/ml RNasin; Promega) at pH7.4. The homogenate was centrifuged at 1000 *g* for 10 min at 4°C, and the resulting supernatant (S1) was subjected to a discontinuous Percoll–sucrose gradients (3, 10, 15, and 23% Percoll from GE Healthcare, prepared in 320 mM sucrose, 1 mM EDTA supplemented with 0.25 mM DTT), centrifuged at 32000 *g* for 10 min at 4°C in a SW41 rotor. The material located at the 10–15% interface was collected, washed for 8 min at 4°C at 12000 *g* in 320 mM sucrose, 5 mM Hepes pH7.4. The resulting pellet was resuspended in an Optiprep Working Solution (OWS) (50% Optiprep [Sigma], 65 mM sucrose, 10 mM HEPES) and separated on an Optiprep-sucrose gradient (9, 12.5, 15, 25, and 35% Optiprep). Optiprep solutions were prepared by diluting the corresponding volume of OWS in 300 mM sucrose, 10 mM HEPES, pH 7.4. Finally, the material located at interface 15–25% was collected and used for RNA isolation. RNA was purified using the PureLink RNA Mini Kit (Invitrogen) according to the manufacturer’s protocol. 50 ng of RNA were retro-transcribed using the Quantitect kit (Qiagen) according to the manufacturer’s protocol. cDNAs were subjected to quantitative PCR on a LightCycler apparatus using the LC480 kit (Roche) according to the manufacturer’s protocol. Each sample was made in triplicate and controls without retrotranscription were routinely added to the PCR. In each experiment, an internal standard was added and quantities of cDNAs were calculated taking the efficiency of each primer into account.

Results are presented as levels of mRNA in SN over quantities in S1, representing the synaptic localization index (I). Results were normalized to HPRT, a transcript restricted to the soma of neurons, with between 0,1 and 5% of total mRNA being found in SN preparation.

Primers used are (5′ to 3′ orientation; fwd =  forward; rev = reverse): CaMKIIα-fwd GCTTTCAGCCAGAGATCACC; CaMKIIα-rev ATGGAGTCGGACGATATTGG; PSD95-fwd AACAGAGGGGGAGATGGAGT; PSD95-rev AAAGATGGATGGGTCGTCAC; HPRT-fwd AGCAGGTGTTCTAGTCCTGTGG; HPRT-rev ACGCAGCAACTGACATTTCTAA.

### Synaptosome Preparation for Metabolic Labeling and Immunoblotting

The previous protocol of SN preparation leads to collection of SN in an Optiprep solution. This made the pelleting of SN difficult, especially since the quantity of material was very low after the 2 gradients. For protein analysis, we thus used an alternate protocol derived from [46]. The SN obtained with both protocols were comparable, since the measured CaMKIIα mRNA synaptic localization was similar and similarly increased by olfactory enrichment.

Briefly, the OBs from 10 adult mice per condition were homogenized in 7 ml of Homogeneization Buffer (TpH) consisting in 320 mM sucrose, 4 mM Hepes pH 7.4, supplemented with protease and RNase inhibitors. The homogenate was centrifuged at 1000 g for 6 min at 4°C. The resulting supernatant (S1) was centrifuged for 10 min at 12500 g at 4°C. The pellet was resuspended in 1 ml TpH, layered on top of a discontinuous sucrose gradient (0.8 and 1.2 M) and centrifuged for 1 h10 min at 16000 rpm at 4°C in a SW41 rotor. The synaptosome rich 1.2M/0.8M interface was collected, pelleted and resuspended in 100 µl Synaptosome Buffer (SnB) containing 10 mM Tris pH 7.5, 2.2 mM CaCl_2_, 0.5 mM Na_2_HPO_4_, 0.4 mM KH_2_PO_4,_ 4 mM NaHCO_3_ and 80 mM NaCl, supplemented with 100 µg/ml chloramphenicol and protease inhibitor. After preincubation at 37°C for 10 min, SN were incubated for 45 min at 37°C with 50 µCi of EasyTag Express Protein Labeling Mix (Perkin Elmer) with or without 10 µM glutamate and 50 µM NMDA. Samples were pelleted, and subjected to SDS-PAGE. Gels were dried and radioactive proteins were detected with a Biorad Personal Molecular Imager FX System.

For western blotting, antibodies were diluted in a solution of 1∶1 PBS:Licor blocking buffer (1∶1) with 0.1% Tween and 0.01% SDS at the following dilutions: CamKIIa 1∶2000 (mouse, Thermoscientific), βactine 1∶4000 (mouse, Sigma-Aldrich), IRDye800 anti-rabbit 1∶10000 (Rockland), IRDye700 anti-mouse 1∶10000 (Rockland). Blots were scanned and quantified using LiCor Odyssey infrared scanning system.

### Drug Treatments and Olfactory Enrichment Protocol

For olfactory enrichment, 10 mice were housed at least 1 h in a clean cage, and presented with a teaball containing either a cocktail of spices (Garlic and Tarragon; Ducros) (enriched group) or nothing (basal group). SN were prepared after 15 min, 30 min or 60 min of exposure to new odours. (*RS*)-3-(2-Carboxypiperazin-4-yl)-propyl-1-phosphonic acid (CPP; Tocris) was used as an NMDA antagonist, at 10 mg/kg injected intraperitoneally (ip) 30 min prior to the 30mn-olfactory enrichment protocol. (*R*)-4-Amino-3-isoxazolidone (D-cycloserine; Sigma) was used as an NMDA agonist at 20 mg/kg ip injection 30 mn before SN preparation.

### Olfactory Habituation/Dishabituation Task

To assess basic olfactory function, mice were submitted to a habituation/dishabituation task in a clean standard home cage. A test session consisted of one 50sec presentation of plain mineral oil (MO) and then four 50 sec odor presentations of the habituation odor (inter trial interval  = 5 min) (Hab 1–4) followed by one 50 s presentation of test odor (Test). Investigation time was measured as active sniffing within 1 cm of the odorant stimuli. A decrease in odor investigation across the habituation trials indicated that the animals detected the odor and remembered it on a short-term basis, from one trial to another. An increase in investigation time upon the presentation of the test odor indicated that it was discriminated from the habituation odor [47].

Odorants used were Pentanol and Carvone diluted to 1 Pa in mineral oil. Each odorant of the pair was randomly used as habituation odor or test odor. Odorants were presented by placing 100 µL of the odor stimulus onto a polypropylene swab in a tea ball hanging from the ceiling of the cage.

#### Data analysis

Investigation time was averaged within groups for each trial. The data were analysed by one-way ANOVA repeated measures across habituation trials to assess habituation and by t-tests between the last habituation trial (Hab4) and the test trial to assess discrimination using Systat statistical software. Results are presented as group mean **±** sem. The level of significance was set to 0.05.

### Associative Learning

To assess the learning abilities of the animals, mice were submitted to an olfactory conditioning.

#### Experimental setup

The mice were tested on a computer-assisted 2-hole board apparatus (40×40 cm) as previously described [33]. The trial started by placing the mouse on the board facing the holes (3 cm diameter, 4.5 cm deep). Latency and sequence of holes visited were measured by a specific homemade software. Holes contained a polypropylene swap impregnated with 20 µL of +limonene (purity 97%, Sigma-Aldrich, Saint-Louis, MO, USA) or mineral oil. Prior to the olfactory learning experiments, the mice were deprived of food (−20% daily consummation, leading to a 10% reduction in body weigh) starting 5 days before shaping.

#### Shaping

During the shaping (12 trials with 15 min between each trial) the mice were trained to retrieve a reward (a small piece of sweetened cereal, Kellogs, Battle Creek, MI) by digging through the bedding with no odor. The reward was first placed on the top of the bedding and after several retrievals, the reward was buried deeply. The shaping was considered to be complete when a mouse successfully retrieved a reward that was deeply in the bedding with a score of 80% correct choices (8 to 12 trials).

#### Conditioning

Conditioning consisted of 24 trials of 2 min with 15 min inter-trial interval [34]. For half of the mice, the reward is systematically associated with +Limonene. The position of the reinforced hole was randomized to avoid spatial learning. The other hole contained no odor or reward.

#### Data analysis

A successful trial was recorded when the mouse first visited the odorized hole (nose poking). For each behavioural session, mean success rates was calculated on blocks of 4 trials and averaged between groups. Mean latencies (time to find the reward) were calculated for each trial and averaged within groups. Results are presented as group mean **±** sem and analyzed using ANOVA (Systat).

### Electron Microscopy

Tissue prepared for electron microscopy followed previously described procedures [48]. Briefly, mice were perfused with 0.1 M PBS containing 1 unit/mL heparin, followed by cold fixative containing 4% PFA and 2% glutaraldehyde in 0.1 M PBS. Following dissection brains were postfixed for 4 h at 4°C prior to sectioning coronally on a vibratome (50 µm). Sections were incubated in 2% osmium tetroxide for 1 h, then dehydrated and stained with 1% uranyl acetate in 70% ethyl alcohol for 1 h. The tissue was embedded in EPON and thin 70–100 nm, sections were cut to include the external plexiform layer (EPL). Ribbons of thin sections were examined on a JEOL 1200 transmission electron microscope and photographed at a primary magnification of 10,000X.

To quantify the frequency of ribosome clusters and dendrites, we used 80 µm^2^ fields from electron micrographs taken at a primary magnification of 10,000X. Within the field, we counted all of the ribosome clusters (a minimum of 2 ribosomes was required) that occurred within GCs dendrites (recognized by their relatively electron dense appearance and irregular contours). We then counted all of the synapses within the same field, including both asymmetrical (mitral cell dendrite to granule spine) and symmetrical (granule cell dendritic spine to mitral cell dendrite).
